# Difficult Digestion Due to Displacements

**Published:** 1900-03

**Authors:** 


**Difficult Digestion due to Displacements.** By A. Symons Eccles, M.B. Pp. 138. London: Baillière, Tindall & Cox. 1899.

The displacements here concerned are not those of the uterus, so commonly described, but the conditions known as enteroptosis and gastroptosis, with a chapter on movable kidney, and another on prolapse of the sigmoid flexure. The author rightly attaches considerable value to the practice of auscultatory percussion in the estimation of the size of a dilated or displaced stomach; and he considers that "the treatment of enteroptosis appears to be almost as important as that of hernia; for in some cases the existence of visceral displacements may lead to the more dangerous conditions of hydro-nephrosis, as in movable kidney; volvulus and intussusception in the small intestine, and possibly cancer in the large bowel; while in gastroptosis there is always a risk of gastrectasia."

It is satisfactory to observe that the numerous cases reported by the author have all been much improved by simple methods of treatment, such as rest in bed, suitable diet, and massage; the maintenance of the general nutrition of the body, and particularly the muscular tone of the abdominal walls, have usually sufficed; and even in the cases of movable kidney, the padded belt has usually kept the kidney in place, so that more active surgical intervention has not been necessary.

